# An In Situ, Child-Led Intervention to Promote Emotion Regulation Competence in Middle Childhood: Protocol for an Exploratory Randomized Controlled Trial

**DOI:** 10.2196/28914

**Published:** 2021-11-09

**Authors:** Petr Slovak, Brett Q Ford, Sherri Widen, Claudia Daudén Roquet, Nikki Theofanopoulou, James J Gross, Benjamin Hankin, Predrag Klasnja

**Affiliations:** 1 Department of Informatics King's College London London United Kingdom; 2 Psychology Department University of Toronto Toronto, ON Canada; 3 Committee for Children Seattle, WA United States; 4 Department of Psychology Stanford University Stanford, CA United States; 5 Department of Psychology University of Illinois Urbana Champaign Champaign, IL United States; 6 School of Information University of Michigan Ann Arbor, MI United States

**Keywords:** randomized controlled trial, children, emotion regulation, in situ intervention, intervention, emotion, protocol, exploratory, efficacy, model, prevention, treatment, risk factor

## Abstract

**Background:**

Emotion regulation is a key transdiagnostic risk factor for a range of psychopathologies, making it a prime target for both prevention and treatment interventions in childhood. Existing interventions predominantly rely on workshops or in-person therapy-based approaches, limiting the ability to promote emotion regulation competence for children in everyday settings and at scale. Purrble is a newly developed, inexpensive, socially assistive robot—in the form of an interactive plush toy—that uses haptic feedback to support in-the-moment emotion regulation. It is accessible to children as needed in their daily lives, without the need for a priori training. Although qualitative data from previous studies show high engagement in situ and anecdotal evidence of the robot being incorporated into children’s emotion regulation routines, there is no quantitative evidence of the intervention’s impact on child outcomes.

**Objective:**

The aim of this study is to examine the efficacy of a new intervention model for child-led emotion regulation—Purrble—that can be deployed across prevention and treatment contexts.

**Methods:**

Overall, 134 children aged 8 to 10 years will be selected from an *enriched* nonclinical North American population; for inclusion, the cutoff for the parents’ rating of child dysregulation will be ≥10 points in the total difficulties score on the Strengths and Difficulties Questionnaire. This cutoff was selected to obtain a measurable, but not necessarily clinical, level of the child’s emotion regulatory difficulties. The selected families will be randomly assigned with .5 probability to receive either a Purrble or an active control (noninteractive plush toy). The primary outcome will be a daily ecological momentary assessment measure of child emotion regulation capability (as reported by parents) over a period of 4 weeks. Exploratory analyses will investigate the intervention impact on secondary outcomes of child emotion regulation, collected weekly over the same 4-week period, with follow-ups at 1 month and 6 months postdeployment. Quantitative data will be analyzed on an intent-to-treat basis. A proportion of families (approximately 30% of the sample) will be interviewed after deployment as part of the process analysis.

**Results:**

The study is funded by the UKRI Future Leaders Fellowship (MR/T041897/1) and an in-kind contribution from the Committee for Children. This study received ethical approval from the Pearl institutional review board (#18-CFC-101). Participant recruitment started in February 2021, with the 1-month deployment in April-May 2021. The results of this analysis will be published in 2022.

**Conclusions:**

This study will be the first quantitative evaluation of the efficacy of an innovative, proof-of-concept intervention model for an in situ, child-led emotion regulation intervention. Insights into the trajectory of daily changes, complemented with weekly questionnaire batteries and postdeployment interviews, will result in an in-depth understanding of whether and how the hypothesized intervention logic model works, leading to further intervention optimization.

**Trial Registration:**

ClinicalTrials.gov NCT04810455; http://clinicaltrials.gov/ct2/show/NCT04810455

**International Registered Report Identifier (IRRID):**

PRR1-10.2196/28914

## Introduction

Maladaptive emotion regulation in childhood is associated with an increased incidence of both internalizing and externalizing mental health disorders [1-4]. In contrast, adaptive emotion regulation in childhood is associated with better mental [5-7] and physical health [8-10]. For these reasons, emotion regulation in childhood is a crucial target for treatment and prevention programs to reduce the societal and personal burden of mental health disorders [11,12].

Although emotion regulation (ER) skills are malleable, and a range of predominantly adult-focused interventions have started to appear in clinical settings (eg, Emotion Regulation Therapy [13] and the Unified Protocol for Transdiagnostic Treatment of Emotional Disorders [14,15]), existing work shows that children’s ER skills are difficult to shape and maintain without detailed guidance and support [16-18]. This work has also shown that parenting strategies play a key role in shaping and maintaining children’s patterns of ER [19-27], but requiring parents’ involvement in existing training, such as in-person workshops, represents yet another barrier to treatment because of well-known issues with access, reach, and cost of parent training programs.

However, the field lacks evidence-based intervention mechanisms to deliver cost-effective ER interventions for children directly in situ, relying instead on extensive in-person workshops (prevention context) or clinical sessions for children and parents (treatment context). Existing approaches thus lead to high costs that disproportionately disadvantage underprivileged families, who would likely benefit most; such families face access- and time-based challenges to take part in available intervention programs [28], although children from low socioeconomic status populations are at risk of low emotion-regulation competencies already at an early age [29], and the gap further widens over the school years [30].

To address the challenges outlined, we developed a proof-of-concept intervention platform to deliver in situ support for child ER during everyday emotionally charged situations, such as the child feeling angry, anxious, or sad. On the basis of a 2-year-long development [31,32], we worked with children, parents, and prevention science experts to co-design an intervention that would support children in strengthening their ER skills. The research prototype was then produced by the Committee for Children (a US-based nonprofit developer of socioemotional learning programs) and Sproutel [33], resulting in a commercial-grade therapeutic toy called Purrble [34].

The initial research prototypes were designed as the first instantiation of a novel *situated* intervention model, which is delivered through an interactive, socially assistive robot sent home with the child or used in schools, without any previous training for either the child or their parent or caregiver. As such, the psychological effects of Purrble are assumed to arise from repeated *bottom-up* support in situ, instead of relying on the traditional *top-down* training contexts delivered through workshops or therapy sessions. In particular, the intervention logic model relies on a 3-stage approach: (1) enabling the child to downregulate emotional moments in situ can (2) provide a preferred alternative to maladaptive emotion regulatory strategies (eg, rumination or suppression) and, over time, (3) lead to shifts in child ER competence [31]. For a detailed description of the hypothesized mechanisms and their links to the intervention design choices, see the *Intervention* section.

To date, 2 qualitative deployment studies have investigated the engagement and acceptability of the prototype in young children’s homes, as well as subjective indicators of effects on emotion regulatory practices (whether positive or negative), as reported by parents and children [31,32]. Findings from these studies have been very positive: all 25 children engaged with the prototype throughout the deployments, all wanted to keep it for longer, and all described how they naturally incorporated it into their everyday routines and gravitated toward it when they needed to downregulate their emotions, including anger, anxiety, or just needing to relax.

Although these early data are promising, we lack quantitative data on the impact of the intervention on child outcomes. In particular, evidence is needed to (1) evaluate the efficacy of Purrble in delivering measurable changes in emotion regulatory practices of children over time and (2) start validating the hypothesized intervention logic model. This study aims to fill these evidence gaps.

## Methods

### Study Design and Objectives

The objective of this study is to evaluate the impact of having access to the Purrble intervention, compared with an active control in the form of a noninteractive plush toy, on child daily ER (primary outcome) as well as a range of secondary outcomes over 1 month.

**Figure 1:**
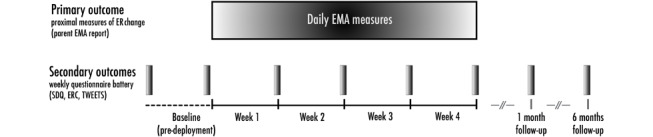
Assessment design. EMA: ecological momentary assessment; ERC: Emotion Regulation Checklist; SDQ: Strengths and Difficulties Questionnaire; TWEETS: Twente Engagement With eHealth Technologies Scale.

**Figure 2:**
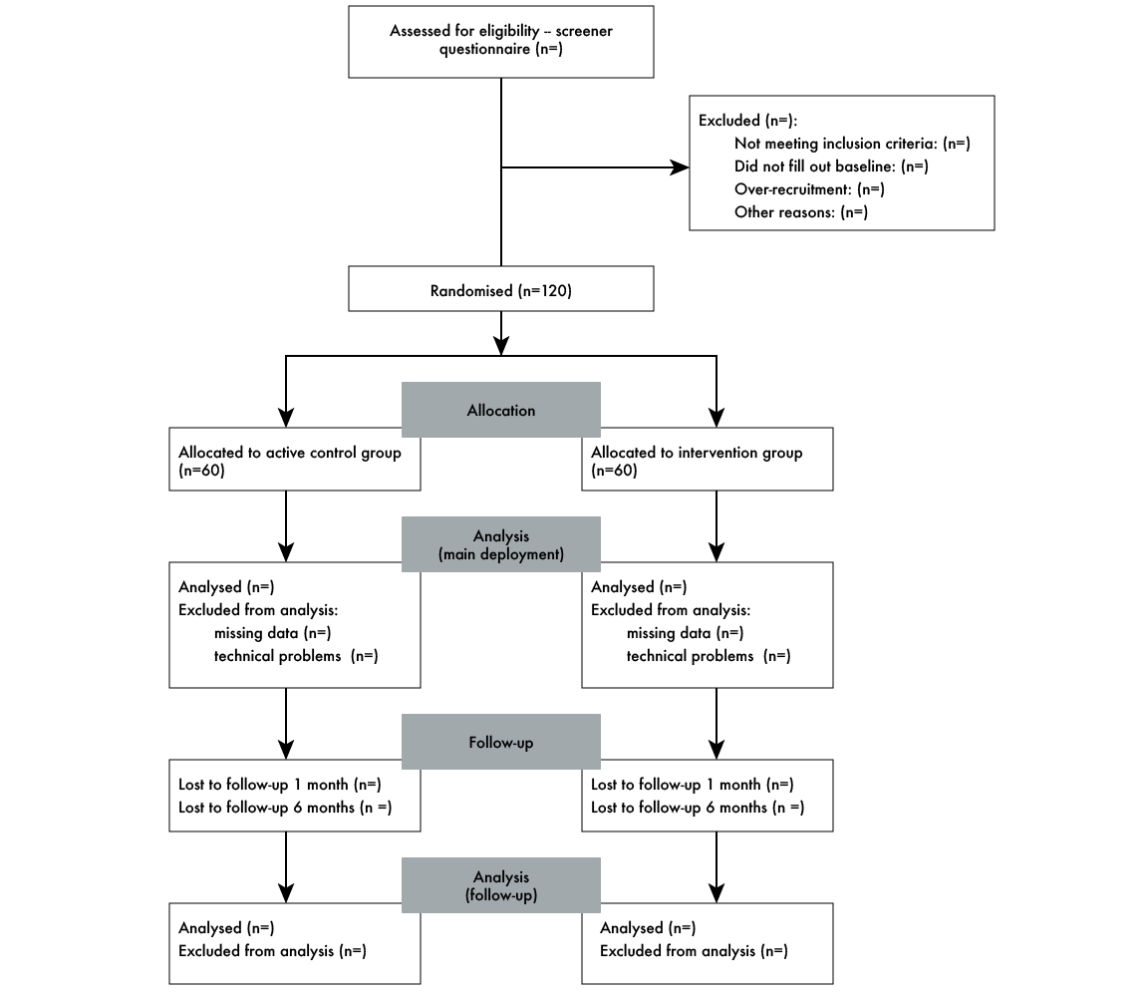
Participant selection flowchart.

The study is a 2-arm, exploratory randomized controlled trial comparing an intervention group (Purrble) with an active control group (noninteractive plush toy). The deployment period will be 4 weeks and will include daily parent self-report measures via ecological momentary assessment (EMA), as well as weekly validated surveys with a 1-month and 6-month follow-up (see Figure 1). The intervention period will start immediately after children receive their arm-appropriate toys. Participants in both the intervention and active control groups (Figure 2) will be able to keep the toys after the deployment period ends. Active control group participants will not be offered Purrble units postdeployment, as this would unblind the conditions before follow-up data collection.

### Intervention

We hypothesize that engagement with an in situ, *bottom-up* ER intervention that enables in-the-moment soothing for children will lead to measurable changes in child self-regulatory behaviors over time.

#### Purrble—Intervention Design and Logic Model

**Figure 3:**
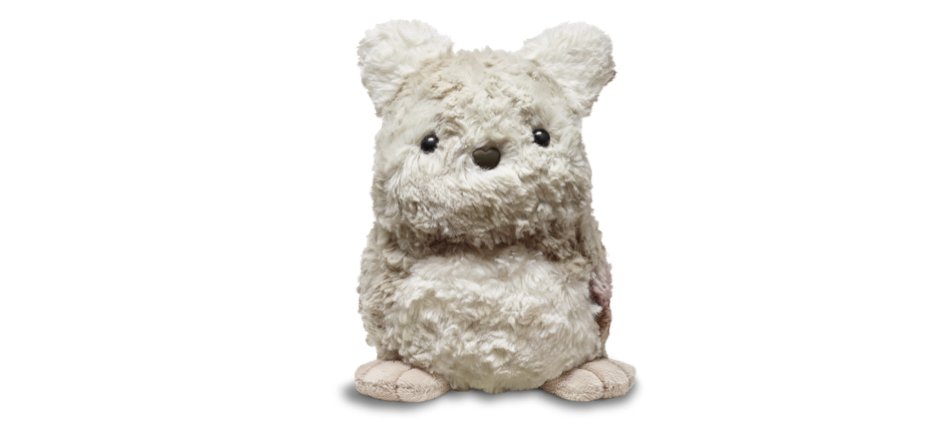
Purrble plush toy.

The intervention takes the form of an interactive plush toy (Figure 3), which was designed to be handed over to the child and support in-the-moment soothing (see Theofanopoulou et al [31] and Slovak et al [32] for the design and data from previous deployments).

The toy is introduced to the child as an anxious creature that needs attention from humans, such as soft stroking and hugging. Embedded electronics enable the toy to produce vibration patterns that simulate a heartbeat (ranging from frantic to slow and steady). When picked up, the toy emits a frantic heartbeat that slows down if the child uses calm stroking movements, as registered by the embedded sensors. If the toy is soothed for long enough, the prototype transitions into a purring vibration indicating a calm, contented state. The minimum time for this transition is less than 1 minute, but the transition can take longer depending on the child-specific interactions with the prototype.

The logic model underlying the intervention is assumed to operate on 3 levels building on each other—see the study by Theofanopoulou et al [31] for more details:

- Level 1 pertains to *directly providing in-the-moment soothing support to children in naturally occurring emotional moments* when they would attempt to calm down. The toy’s physical and interaction design was aimed at tapping into various known regulatory factors, grounded theoretically in the Gross extended process model of ER [35]. Specifically, we designed the prototype interaction with the aim to impact 2 separate stages of the ER process: the *attentional deployment stage* [36-39], by shifting children’s attention from the emotion-eliciting situation toward interacting with the toy, and the *response modulation stage*, by facilitating down-regulation through pleasant tactile interaction analogously to the mechanisms presumed to underpin emotion regulatory effects of human-animal interaction [40-45].
- Level 2 is concerned with mechanisms that *facilitate children’s long-term engagement* with the intervention, building on the positive subjective experience of in-the-moment soothing. The framing of the toy as an *anxious creature in need of assistance* is the hypothesized key driver; we assume that this will not only frame the interactions regarding helping regulate others’ emotions (extrinsic ER; [41,46,47]) but also facilitate the creation of a sense of relationship and responsibility for the well-being of the creature, similar to the long-term engagement seen with child-orientated robots [48] or products such as Tamagotchi [49-51].
- Finally, level 3 is assumed to emerge from repeated experiences of soothing interactions over time, leading to a *shift in children’s ER practices and implicit beliefs about emotion* (ie, the individual’s beliefs about whether emotions can be regulated; see study by Ford and Gross [52] for details). Specifically, we hypothesize that repeated interactions with the toy will result in a shift in children’s implicit beliefs about the controllability of emotion [52,53], a well-known target for intervention [54-58], as well as help reduce maladaptive ER patterns such as rumination or suppression [59].

Deployments with the research prototypes underpinning the current Purrble [31,32] show that, across all 25 families, children reported that the smart toy was incorporated into the children’s ER practices and engaged with naturally in moments the children wanted to relax or calm down. Specifically, the data from [31] shows that the children interacted with the toy throughout the week-long deployment (eg, average active use for 74.9, SD 64.1 minutes per day; median 60.5), they found the experience enjoyable, and all children requested to keep the toy longer. Children’s emotional connection to the toy appears to have driven this strong engagement. Parents reported satisfaction with and acceptability of the toy. No quantitative data on changes in ER were collected in previous studies because of the small sample size and the focus on feasibility and understanding of appropriation within families.

#### Active Control Group

When compared with a traditional noninteractive plush toy, the intervention model underpinning Purrble includes 2 possible pathways through which the effects should occur:

- The first is the in-the-moment soothing support (level 1 in the logic model) that we hypothesize is driven by the interactivity of the toy. The lack of such situated down-regulation support should thus be the key difference between the intervention and an active control, that is, a noninteractive stuffed toy, leading to lower engagement over time (level 2) and a lack of impact on child ER practice (level 3).
- However, an alternative pathway is the ER routines (levels 2 and 3 in the logic model) that could, in principle, emerge around the intervention narrative of a physical object to use for calming down, even without the toy being interactive. In other words, if it was simply the narrative of an anxious creature in need of care (rather than the combination of the narrative together with the interactivity) that drives long-term engagement and changes in behavior, a noninteractive stuffed toy could still lead to the development of the same routines. We see this as an unlikely scenario, for example, given the prevalence of plush toys in most, if not all, households—but one that should be addressed in the study design.

For these reasons, we argue that a comparison with a nonactive control—such as waiting list or treatment-as-usual (ie, nothing)—would not allow us to distinguish the hypothesized impact on in-the-moment soothing of interactivity versus the emergence of new family routines and would also be open to unequal social desirability bias. However, from the perspective of the hypothesized logic model, it is not necessary for the active control to have exactly the same form factor as the active toy, as long as it is comparable in size, shape, and appeal. In fact, we have explicitly decided not to use deactivated Purrble units as active controls because of the increased risk of unblinding, whereby the participants search for or come across Purrble on the web (or notice the plastic enclosure with electronics inside the toy) and assume that their unit is malfunctioning.

#### Selection and Validation of Active Control Units

The selected active control toy is the *Wild Republic 8″ Hedgehog animal*. The selection process was guided by the following requirements: the plush toy needed to have analogous size, weight, and quality of materials, and at least similar (if not higher) visual appeal. We also made sure to include the design characteristics that our previous work suggested were important for the narrative around the toy [31,32]. These included selecting a similarly stylized animal (to enable emotion projection and feelings of care), as well as no visible mouth on the toy (to prevent setting an expectation about the toy’s emotional state as a mouth would imply an emotional expression). In addition, we have adapted the one-page parent-facing descriptions of the narrative that come with Purrble also for the active control unit; as such, the active control families will receive the same general narrative—including that the creature is anxious and needs human care, but without the explicit mentions of the toy interactivity—and the same suggested activities for parents.

**Figure 4:**
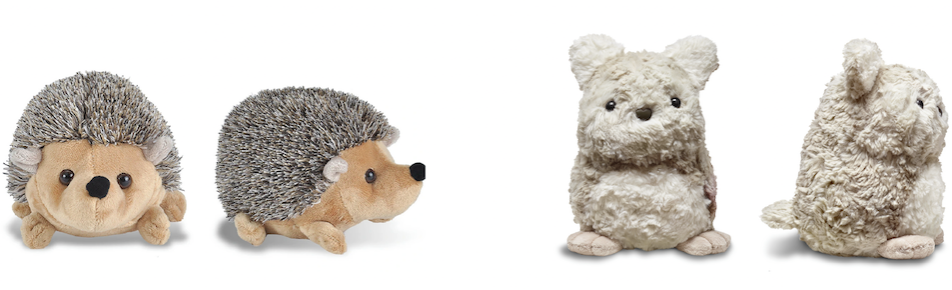
On the left, an image of the Hedgehog toy for the active control group. On the right, an image of Purrble for the intervention.

To validate that the active control is at least as visually appealing as the intervention, we ran a web-based experiment in which participants were randomly assigned to rate either the Hedgehog or the Purrble images. In both cases, the prompts were professional photos from the front and side on a white background, presented at equal size (Figure 4). The experiment was powered to detect a medium-sized effect (*d*=0.4) at 80% power for a comparison on a single measure, resulting in a sample size of 200 (1:1 allocation ratio). Participants were recruited through the web-based research platform Prolific, with the survey hosted by Qualtrics (including blocked randomization). Inclusion criteria for parents were the eldest child born in 2010-2013 (approximately aged 8-10 years), country of residence in the United Kingdom or the United States, and above 95% acceptance of tasks on Prolific. The participants were prompted to imagine that their oldest child had received the plush toy pictured above as a present. We then asked 3 questions, with the first question—appeal—preselected as the primary measure: (1) How appealing do you yourself find the toy? (2) How appealing do you think your child would find the toy? and (3) How likely would you be to recommend this toy to another parent?

**Figure 5:**
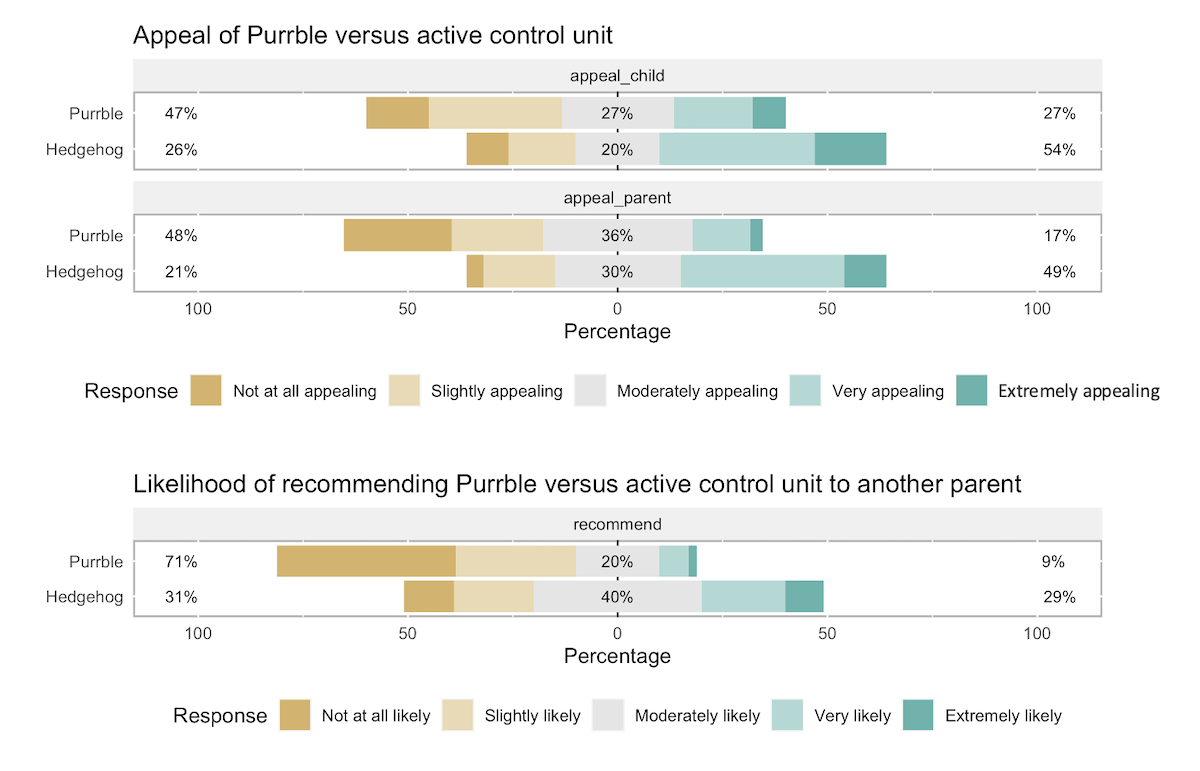
Rating of the questions regarding the appeal of Purrble versus the active control unit, and the likelihood of recommending Purrble versus the active control unit to another parent.

The results show that the hedgehog was consistently rated higher than Purrble on all 3 questions (Figure 5). This suggests that, if anything, the active control should be more appealing to our study participants than the intervention units: it is a particularly stringent control condition to test the effect of a visually appealing, but noninteractive, plush toy. In other words, if it was purely the visual appeal of the units that would drive child or parent engagement and the resulting ER effects (as opposed to the interactivity of the intervention units), we would expect the active control to show at least as good if not better engagement and reports of changes in child ER from the families.

### Participant Eligibility Criteria

Given Purrble’s intended use as a targeted prevention intervention, we will recruit an *enriched* population of neurotypical children, aged 8-10 years, from families in the United States. The enrichment consists of recruiting families where the child is seen as struggling with some level of ER difficulty (as reported by their parent) but is not undergoing clinical treatment.

The specific inclusion criteria were a child aged 8-10 years, parent-reported score of ≥10 for the total difficulties score on the Strength and Difficulties Questionnaire (SDQ). The exclusion criteria for the child were current participation in another mental health intervention. In addition, an exclusion criterion for the parent and/or the child is not being fluent in English (as all measurement scales are in English).

The target child age range has been selected as collecting self-report measures from young children is a well-known challenge, especially when inquiring about complex cognitive concepts such as those involved in ER [60,61], and our pilot work in families and schools suggests that acceptability for the toy is high with children of this age, with children aged 8-10 years still using the toy as intended.

### Recruitment, Randomization, and Blinding

Parents will be recruited by sending the study invitation to a mailing list of approximately 10,000 parents or guardians, whose children are receiving Committee for Children programs in school, and who have signed up to receive information from the Committee for Children.

Once eligibility is proven and parents fill out the baseline questionnaires, families will be randomly assigned to the intervention or active control group. Families will be randomized using a computerized algorithm and randomly permuted block sizes. The allocation schedule is generated by a Committee for Children researcher not directly involved in the data collection (and not aware of participants’ details apart from their address) and is unknown to the investigator and the participants. The principal investigators will not be aware of the allocation until data collection is complete.

Blinding is present: the families will not be aware of the existence of another condition throughout the study, requiring the participation cap on students from any single class.

### Outcome Measures

The primary outcome will be a daily EMA measure of child emotion regulation capability (as reported by parents) over a period of 4 weeks. Exploratory analyses will investigate the intervention impact on secondary outcomes of child emotion regulation, collected weekly over the same 4-week period, with follow-ups at 1 month and 6 months postdeployment. See Table 1 for a summary of the outcome measures and assessment times.

**Table 1:** Summary of the outcome measures and assessment times.

Outcome measure	Baseline	Deployment				Follow-up			
		Week 1	Week 2	Week 3	Week 4	1 week	2 weeks	1 month	6 months
Perceived child ER ability		Collected daily	Collected daily	Collected daily	Collected daily				
Daily parent report ecological momentary assessment (modified differential emotions scale)		Collected daily	Collected daily	Collected daily	Collected daily				
Daily parent report ecological momentary assessment (engagement)		Collected daily	Collected daily	Collected daily	Collected daily				
Daily parent report ecological momentary assessment (reaction to triggers)		Collected daily	Collected daily	Collected daily	Collected daily				
Weekly parent questionnaire (Strength and Difficulties Questionnaire)	✓	✓	✓	✓	✓			✓	✓
Weekly parent questionnaire (ER Checklist)	✓	✓	✓	✓	✓			✓	✓
Weekly parent questionnaire (Twente Engagement With eHealth Technologies Scale)		✓	✓	✓	✓			✓	✓
Weekly children questionnaire (Difficulties in ER Scale)	✓	✓	✓	✓	✓			✓	✓
Weekly children questionnaire (emotion regulation-beliefs)	✓	✓	✓	✓	✓			✓	✓
Interviews with families						✓	✓		

#### Primary Outcome Measure

The primary outcome measure will consist of a composite end-of-day 4-item parent report measure of the *perceived child ER* ability throughout the day. The specific items are listed below, all measured as a visual analog scale [62,63] with *not at all* and *very much so* as the anchors. The composite score for each day will be computed as the mean value across the 4 items:

1. Today, to what extent was your child able to take difficult things in a stride?
2. Today, to what extent did your child get easily triggered or upset? (reverse-scored)
3. Today, to what extent was your child able to calm down easily if upset?
4. Today, to what extent did your child get very emotional even after the littlest things? (reverse-scored)

Specifically, this EMA item composite aims to indirectly tap into day-to-day changes in a child’s ability to downregulate their emotions after (and thus cope with) triggering situations they routinely experience in their daily life. Our expectation is that contact with the Purrble will lead to the following:

1. Lower intensity negative emotions after facing everyday stressors (eg, by being able to downregulate before emotional response escalate—cf, level 1 in the theory of change).
2. Briefer duration of negative emotions after facing everyday stressors (eg, being able to downregulate emotions faster with Purrble—cf, levels 1 and 3 in the theory of change).

By being measured repeatedly over time and within subjects, the items capture the changes in emotional outcomes that would indicate changes in the child’s ER ability, assuming that the trait-based reactivity to daily stressors remains stable. The items were thus selected to tap into the proximal outcomes of ER behavior that (1) would be affected if the intervention is effective, (2) is directly observable by parents, (3) is state-based (rather than trait-based) to enable daily measurement, and (4) is connected to the intervention theory of change.

The item selection drew on a range of established measures of emotion dysregulation (Strengths and Difficulties Questionnaire, SDQ [64], Emotion Regulation Checklist, ERC [65], Difficulties in Emotion Regulation Scale (DERS) [66], and Children’s Emotional Management Scales [67]), as well as qualitative data from previous deployments [31,32] with parental reports of increased emotion regulation after potentially triggering events being the common theme.

#### Secondary Outcome Measures

##### Daily Parent Report EMA

In addition to the primary EMA outcome, we will collect several other daily EMA parent reports. The psychological constructs targeted are the *child’*s *general mood* and *daily engagement with the toy.*

The *child’s daily mood* is measured by selected Modified Differential Emotions Scale (mDES) [68] emotion triplets, balancing 2 negative and 2 positive sets, while being informed by previous qualitative studies. The items are listed as follows, all measured on a visual analog scale [62,63], with *not at all* and *extremely* as the anchors:

1. How stressed, nervous, or oevrwhelmed did your child feel today?
2. How joyful, glad, or happy did your child feel today?
3. How angry, irritated, or annoyed did your child feel today?
4. How proud, confident, or self-assured did your child feel today?

The *daily engagement item* asks about the general perception of engagement with the toy (*How much did your child play with the toy today?*)*,* measured on a visual analog scale [62,63], with *not even once* and *they were inseparable* as the anchors.

Finally, we will include a *series of explorative items that examine the child’s reaction after potentially triggering events*. We will first ask the parents *Did anything happen today that would typically upset your child?* If yes, the protocol follows with several questions collecting qualitative and quantitative information regarding the number of such situations, the intensity and length of subsequent children’s negative reactions, how many of these situations the child used the toy, who initiated the use, how helpful or unhelpful it was, and an opportunity to share open-ended comments or observations. The purpose of these items is to gain a qualitative understanding of the toys’ use in challenging situations and to guide post deployment interviews.

##### Weekly Parent Reports

We will also collect *secondary distal outcomes* for both the intervention and active control groups, with 5 data points collected during the 4-week main deployment period: at baseline (just before intervention or control toys are delivered), and then weekly for a period of 1 month (end of week 1, week 2, week 3, and week 4), and then at 1-month and 6-month follow-up. The measures include parent reports on distal outcomes of child emotion regulation (SDQ and ERC) and engagement (adapted Twente Engagement With eHealth Technologies Scale [TWEETS]), as well as child reports on their emotion regulation strategies (DERS) and emotion regulation beliefs (ER mindset).

**Weekly Questionnaires—Parents**

*Parent-reported emotional and behavioral difficulties of the child* will be measured using the 25-item SDQ [64]. This well-established measure has shown satisfactory reliability and validity [64,69] and is commonly used to measure the impact of child-orientated interventions [46,47].

*Parent-reported ER lability and competence* will be measured using the 24-item Emotion Regulation Checklist [65] questionnaire. The ERC measures children’s general emotion regulation capacities and consists of 23 questions divided into 2 subscales, which we will consider separately: the Lability or Negativity subscale measures inflexibility, liability, and dysregulation, whereas the Emotion Regulation subscale measures positive emotion regulation behavior and capacities, appropriate emotional expression, empathy, and emotional self-awareness. ERC is one of the most commonly used measures of emotion regulation in children [5].

The *parent-reported behavioral, cognitive, and affective engagement* with the intervention will be measured using an adapted version of the Twente Engagement with E-health Technologies Scale (TWEETS) [70] questionnaire. TWEETS is a new, promising instrument specifically designed to measure engagement with digital mental health interventions, with good reliability in previous studies [70]. The adaptation here is necessary to track parents’ perceptions of child engagement, rather than the original self-report version. See Multimedia Appendix 1 for the fully adapted instrument.

**Weekly Questionnaires—Children**

*Child-reported emotion dysregulation* will be measured by a shortened version of the brief DERS [71], following previous work with children of similar ages (8-9 years [72]). DERS has been developed to measure clinically relevant difficulties in ER across 6-factor analytically derived subscales (awareness of emotion, clarity about own emotions, nonacceptance of emotion, lack of effective emotion regulatory strategies, lack of ability to engage in goal-directed activities, and lack of ability to manage impulses). The DERS [71,73] has been used extensively to facilitate understanding of how emotion dysregulation is associated with psychiatric symptoms and to measure treatment progress. See Multimedia Appendix 1 for the full adapted instrument.

Child-reported *beliefs about ER* beliefs questionnaire [54] have been adapted to child populations. The questionnaire measures child *entity beliefs* about their emotions [52,55,74], that is, whether children believe their emotions to be controllable. To simplify the required cognitive load, the adapted measure asks children to pick 1 out of 4 statements (eg, *I cannot control my feelings at all, I can control my feelings a little, I can control my feelings a lot,* and *I can control my feelings all the time*) rather than using the original Likert scale statements asking about agreement (eg, *The truth is, I have very little control over my emotions*). Our preliminary validation (221 children, aged 6-10 years, US sample) showed good reliability (0.844) (internal pilot study), compared with the adult version [54]. See Multimedia Appendix 1 for the full adapted instrument.

#### Postdeployment Interviews (Process Analysis)

We will collect semistructured interview data with parents of up to 40% of the experimental group sample (20-25 families), and approximately 25% of the control group (15 families). The interviews will be conducted within 2 weeks following the primary data collection period. We will specifically aim to recruit families who show the highest or lowest change in the outcome data over the primary period to qualitatively understand the potential moderators of intervention responses for future research.

Following previous work [31], the semistructured interview guide will explore the engagement with the toy, any qualitative changes in child or family behavioral patterns that parents notice, appropriation (ie, how the intervention ended up being used by different participants), and use trajectory over time. In addition, we draw on the data from daily questionnaires as part of the interviews, such as discussing the trajectories of daily parental reports on child ER with the parent (eg, asking about specific instances where there is a spike or as a way of referring to particular times in the deployment).

#### Hypotheses

##### Primary Hypothesis

Across the trial, we hypothesize that access to the Purrble intervention (as opposed to the active control) will lead to an *increase in parent-reported daily child ER ability*, as measured by the primary outcome.

##### Secondary Hypotheses

Intervention effects will be moderated by daily engagement with toy and weekly data from the TWEETS questionnaire. In addition, we expect to see between-group differences in favor of Purrble for the secondary daily EMA parental-report outcomes: *an increase* in the *positive mDES items* and a *decrease* in the *negative mDES items*.

Finally, as exploratory analyses, we will investigate the following hypotheses for weekly outcome measures:

1. The decrease in *parent-reported emotional and behavioral difficulties* (as measured by the SDQ) will be greater in the intervention group (smart toy) than in the active control group (noninteractive toy).
2. *The decrease in ER lability* and *increase in emotion regulation competence* (as measured by the Lability or Negativity subscale and Emotion Regulation subscale questionnaires respectively) will be greater in the intervention group than in the active control group (noninteractive toy).
3. *Behavioral, cognitive, and affective engagement* with the intervention, as measured by the adapted *TWEETS questionnaire for parents*, will be higher for the intervention than the control group.
4. The decrease in *child-reported emotion dysregulation* (as measured by the DERS) will be greater in the intervention group than in the active control group (noninteractive toy).
5. The decrease in *child-reported entity beliefs of emotion regulation* (as measured by the ER mindset questionnaire) will be greater in the intervention group than in the active control group (noninteractive toy).

#### Adherence Protocol

We will use the following protocol to encourage participants’ adherence to the data collection schedule. All decision points are based solely on data collection, rather than any indication of the intervention use or nonuse. The protocols for daily and weekly data collection were independently run.

*The daily measures adherence protocol* will be as follows: when a participant misses their end-of-day questionnaire, the system automatically generates a reminder next morning. If a participant has already received 2 automated reminders in a row and again misses daily measures, a research assistant will call the participant (in addition to an automated reminder) on the next workday, following a predetermined call script. If the participant does not respond, they will receive one more reminder the next day, and a second call on the following workday. If no data are received, the participant will be marked as dropped out as they will have missed at least 6 subsequent daily measures (ie, more than 20% of the overall data points). The protocol resets when the participant submits a daily questionnaire. In summary, the daily adherence protocol was as follows: 2× reminder, 1× call+reminder, 1× reminder, and 1× call+reminder, dropped from the study.

The *weekly measures adherence protocol* will be as follows: the survey links will be sent on Saturday midday, with an automated email reminder on Sunday morning. If data are missing by the end of Sunday, a research assistant will call the participant on Monday, following a predetermined call script. The participants will be sent a new link to the survey, with the possibility of submitting their response for the week by the end of Monday. We will not use adherence to weekly surveys as a decision to drop participants from the study, as weekly surveys do not collect the primary outcome.

All calls and other communications with participants will be logged by the research assistant on the web. Although the research assistant will be able to unblind the participants’ condition if necessary, we do not expect this will be needed in most of the calls.

#### Data Analyses

All analyses of daily EMA outcomes (the primary outcome of daily ER and secondary outcomes of engagement with the toy and mood), as well as the analyses of the weekly outcomes, will be conducted using random-effect models for longitudinal regression. These models consider the nested nature of repeated-measures data and are robust to data missingness and violations of the normality assumption. The regression models will examine the difference in the outcome as a function of the assigned condition (Purrble vs active control). The models will adjust for the baseline levels of the score on the total difficulty scale in the SDQ to decrease noise. To account for participant-to-participant variability in EMA scoring as well as in trajectories of change in EMA scores over time, we will include a random intercept and random slope for time. All analyses will be conducted on an intent-to-treat basis and will use data from all randomized participants regardless of their level of participation.

A separate model will be fitted to each outcome. Given that this is an exploratory trial, we will not formally adjust for multiple comparisons. However, we will be cautious in our conclusions about any significant findings and will interpret all results in light of all performed analyses.

To examine the link between engagement with the intervention and ER outcomes, we will conduct 3 types of exploratory analyses: first, given that we hypothesize that Purrble will be more engaging than the control-condition stuffed animal, we will examine whether intervention engagement is influenced by the treatment condition. To do so, we will regress our daily measure of engagement on the treatment condition, controlling for the previous day’s engagement to reduce noise. Similarly, we will regress the weekly TWEETS scores on the treatment condition, controlling for the previous week’s engagement. Second, we will examine whether engagement moderates the intervention impact on both our primary outcome, the daily measure of emotional regulation, and the weekly ERC and SDQ measurements. For the daily model, we will use our primary measure, the EMA assessment of ER, as the dependent variable, and will include, as regressors, treatment indicators and the EMA measure of engagement, as well as a term for their interaction. To decrease noise, the model will also include as a covariate the previous day’s score on the ER measure. Similarly, for weekly models, we will regress the weekly ERC and SDQ scores on terms for the treatment indicator, TWEETS score, and their interaction. As in the daily model, we will also include a term for the ERC or SDQ score of the previous week to reduce noise. As an alternative approach, we will consider using the weekly average of the daily EMA engagement scores rather than TWEETS for the weekly analyses of the moderating influence of engagement, as these 2 scales tap into different aspects of the engagement experience.

Finally, as our logic model postulates that engagement may also mediate the impact of intervention, we will also conduct an analysis of the mediating role of engagement. Given the ambiguities of mediation analyses in longitudinal settings and the lack of consensus on best practices, for this analysis, we will follow the original Baron and Kenny approach to establishing mediation [75]. To do so, we will use, as our outcome variables, the change scores for the emotion-regulation measures (ERC and SDQ) from baseline to the end of study (end of week 4). To measure engagement, we will use the average of the weekly TWEETS assessments over the course of the study. To examine the mediating role of engagement, we will conduct 3 sets of analyses: first, we will estimate the impact of the treatment condition on the change in ER by regressing the emotion-regulation change score on the indicator of the treatment condition. Second, to examine whether treatment had an impact on engagement, we will regress the average of the weekly TWEETS assessments on the indicator for the treatment condition. Finally, we will estimate a model that regresses the ER change score on both the treatment indicator and engagement. To assess preliminary evidence on the mediating role of engagement, we will examine the magnitudes of effect coefficients in all models, as well as statistical significance, to determine whether the inclusion of engagement in the model has reduced the impact of the treatment condition. Given that the ERC and SDQ tap into different aspects of emotion regulation, we will conduct these analyses for both ERC and SDQ change scores.

#### Sample Size and Power

We calculated the sample size requirements to be able to detect a difference between the two arms on our primary outcome measure: the daily parent report of the child’s ER throughout the day. On the basis of the data from our preliminary studies, we expect to see a medium effect size for this measure; therefore, to be conservative, we used a Cohen *d* of 0.3 in our sample size calculation. With this assumption, we calculated the sample size to be able to detect the main effect of the condition (Purrble vs active control) with 90% power and an α level of .05. Under the conservative assumption that the correlation between repeated measures will be 0.75, the required sample size is 92 participants. We inflated this number by 10% to help power the exploratory analyses of the secondary outcomes. Assuming a 20% dropout rate, our final sample size is 120 families.

### Ethical Criteria and Ethics Committee

The study will be conducted according to local regulations and the Declaration of Helsinki. The Pearl institutional review board approved the study (#18-CFC-101). Written informed consent will be obtained from all parents, and written assent will be obtained from all children. The trial is registered with ClinicalTrials.gov (NCT04810455).

## Results

This study is funded by the UKRI Future Leaders Fellowship (MR/T041897/1) and the Committee for Children. The ethical approval was received, and the study is preregistered with ClinicalTrials.gov. The recruitment procedures started in early March 2021. The data collection started in mid-April 2021, with the primary data collection period finished by the mid-May 2021.

## Discussion

### Principal Findings

This study aims to evaluate the benefits of access to a socially assistive robot on children’s ER ability in situ, without the need for training for the child or parents. If successful, the study will provide a proof-of-concept example of a *bottom-up* ER intervention, enabling a new approach to developing child ER competency through technology-enabled ongoing support in everyday emotional situations. As such, this work complements the currently predominant *top-down* approaches, where ER strategies are taught in training contexts, and then the children are expected to transfer these strategies into daily life, often with no or limited in situ support. If shown effective, these in situ interventions can inspire a new approach in how ER interventions can be conceptualized, designed, and delivered.

More generally, ER in childhood is a prime target for a range of prevention and clinical interventions. In this regard, the existing Purrble toys can be seen as a potentially highly modular and extendable platform, where additions or minor changes to the core interaction paradigm can be used to target a range of participants (different ages, verbal acuity, etc), and a variety of different contexts (clinical and nonclinical settings). For example, our pilot data with clinicians and psychotherapists suggest potential benefits in the context of eating disorders and self-harm interventions for adolescents, as well as complementing therapeutic support for fostered or looked-after children. Rigorous empirical data supporting the efficacy of the current intervention are crucial for such research.

### Limitations

The following limitations of this study need to be considered. First, this study is designed as an exploratory RCT to account for the uncertainty about the effect sizes that should be expected given the novelty of the intervention delivery mechanism and proposed theory of change. The range of selected secondary measures (and the substantial qualitative process analysis) reflects this focus on hypothesis generation rather than aiming to design a definitive trial.

Second, a related limitation concerns the choice of primary measure. The bespoke 4-item composite measure of the parent-reported *child emotion regulation* ability throughout the day reflects the uncertainty about the impact of the intervention on distal ER outcomes, aiming to measure time-sensitive proximal aspects of the expected changes in child emotion regulatory ability that are also directly observable by the parent. Future work should target more established measures such as those targeted by the secondary outcome in this study (SDQ, ERC, DERS, and ER beliefs). We expect that the necessary sample size estimation for such studies will be guided by the empirical data collected in this study.

Third, the current commercial Purrble toys lack the capability to track in-the-moment interactions over time or gather any other data on daily use, as do the noninteractive active control units. As such, the study lacks objective measures of daily engagement and needs to rely on observer-report measures (parents), who are unlikely to fully account for the child’s independent use of the toy. In addition, the lack of in-the-moment tracking also limits the methods available to verify level 1 (in-the-moment soothing) and level 2 mechanisms (child-initiated repeated interaction) from the theory of change. If the data from this study show the impact of the Purrble intervention on child ER in situ, future mechanistic or optimization studies should specifically focus on testing the pressumed level 1 and 2 processes.

Finally, the study design relies predominantly on end-of-day parent reports of child ER under naturally occurring daily stressors. This is in line with other studies on similarly aged child samples, and the ecological validity of the findings is a strength of the study design. However, further work could extend these methods with more controlled measures, such as in-laboratory experimental measures, as another triangulation of the meaningful impact of the intervention on child ER.

### Conclusions

The proposed study is an explorative RCT that assessed for the first time the efficacy of a novel intervention model for child-led ER, delivered in situ through an interactive socially assistive robot. The strength of the approach lies in the ecologically valid deployment, with a strong active control condition that limits the effects of social desirability bias. If successful, the robotic platform can serve as a proof-of-concept example for a new approach to ER interventions, shifting the learning support directly into the daily moments when ER competencies need to be applied. Such a *situated* intervention model can be a good complement to the current therapy or workshop-based interventions.

## Appendix

Multimedia Appendix 1 Adapted instruments.

## Abbreviations

CEMS: Children’s Emotional Management Scales
DERS: Difficulties in Emotion Regulation Scale
EMA: ecological momentary assessment
ERC: Emotion Regulation Checklist
ER: emotion regulation
mDES: Modified Differential Emotions Scale
SDQ: Strengths and Difficulties Questionnaire
TWEETS: Twente Engagement With eHealth Technologies Scale
