# Supplementary material for: An In Situ, Child-Led Intervention to Promote Emotion Regulation Competence in Middle Childhood: Protocol for an Exploratory Randomized Controlled Trial
Source: JMIR Res Protoc. 2021 Nov 9;10(11):e28914. doi: 10.2196/28914 (PMC8663453; doi:10.2196/28914)

# Appendix

## Adapted TWEETS questionnaire

**Answer anchors:**5-point Likert scale

(strongly disagree=0, disagree=1, neutral=2, agree=3, strongly agree=4).

**Behaviour**

[this technology] is part of my daily routine

**→ My child uses Purrble every day.**

[this technology] is easy to use

**—> Purrble is easy to use for my child.**

I'm able to use [this technology] as often as needed (to achieve my goals)

**—> My child is able to use Purrble as often as they need to.**

**Cognition**

[this technology] makes it easier for me to work on [my goal]

**—> Purrble makes it easier for my child to learn how to calm themselves down.**

[this technology] motivates me to [reach my goal]

**→ Purrble motivates my child to calm down when they need to.**

[this technology] helps me to get more insight into [my behavior relating to the goal]

**→ Purrble helps my child get more insight into how to calm themselves down.**

**Affect**

I enjoy using [this technology]

**→ My child enjoys using Purrble.**

I enjoy seeing the progress I make in [this technology]

**→ My child enjoys the relationship they have with Purrble.**

[This technology] fits me as a person

**→ Purrble is a good fit for my child's needs.**

## Adapted DERS questionnaire

Please indicate how often the following statements apply to you.

Answer anchors: *Almost Never – Sometimes - About half of the time - Most of the time - Almost Always*

- I pay attention to how I feel.
- When I’m upset, I pay attention to my feelings.
- When I’m upset, I get embarrassed for feeling that way.
- I pay attention to my feelings.
- When I’m upset, I have a hard time doing things.
- When I’m upset, I get out of control.
- When I’m upset, I have a hard time paying attention to other things.
- When I’m upset, I feel bad for feeling that way.

- When I’m upset, I feel guilty for feeling that way.
- When I’m upset, I have a hard time thinking.
- When I’m upset, it is hard to control my actions.

## Adapted ER-beliefs questionnaire

Kids sometimes have different ideas about feelings. Under each number you will find some sentences expressing some of these ideas. Please read each of the sentences carefully and tick the box next to the sentence that describes you best.

There is no right or wrong answer. Just choose the sentence that describes you best. Put a mark like this next to the sentence that you pick. Please tick only one box under each number.


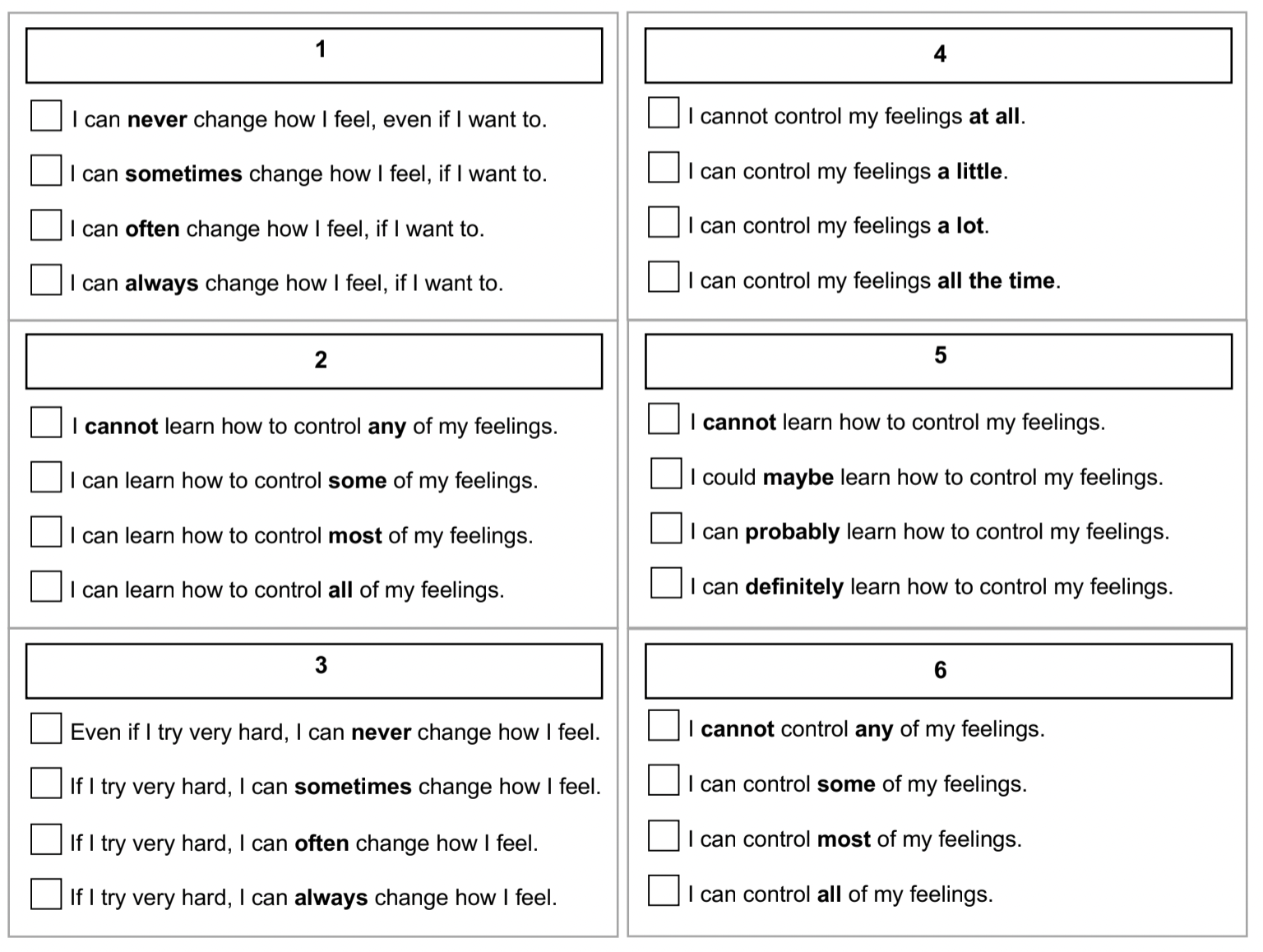

Supplement: Multimedia Appendix 1 [file resprot_v10i11e28914_app1.docx]
